# Land use regression modeling of intra-urban residential variability in multiple traffic-related air pollutants

**DOI:** 10.1186/1476-069X-7-17

**Published:** 2008-05-16

**Authors:** Jane E Clougherty, Rosalind J Wright, Lisa K Baxter, Jonathan I Levy

**Affiliations:** 1 Harvard School of Public Health, Department of Environmental Health, Landmark Center 4th Floor West, P.O. Box 15677, Boston, MA 02215, USA; 2 Channing Laboratory, Brigham & Women's Hospital, Harvard Medical School, Landmark Center, 401 Park Drive, Boston, MA 02215, USA; 3 Harvard School of Public Health; Department of Society, Human Development, and Health, 677 Huntington Avenue, Boston, MA 02215, USA; 4 National Exposure Research Laboratory, U.S. Environmental Protection Agency, Research Triangle Park, NC 27711, USA

## Abstract

**Background:**

There is a growing body of literature linking GIS-based measures of traffic density to asthma and other respiratory outcomes. However, no consensus exists on which traffic indicators best capture variability in different pollutants or within different settings. As part of a study on childhood asthma etiology, we examined variability in outdoor concentrations of multiple traffic-related air pollutants within urban communities, using a range of GIS-based predictors and land use regression techniques.

**Methods:**

We measured fine particulate matter (PM_2.5_), nitrogen dioxide (NO_2_), and elemental carbon (EC) outside 44 homes representing a range of traffic densities and neighborhoods across Boston, Massachusetts and nearby communities. Multiple three to four-day average samples were collected at each home during winters and summers from 2003 to 2005. Traffic indicators were derived using Massachusetts Highway Department data and direct traffic counts. Multivariate regression analyses were performed separately for each pollutant, using traffic indicators, land use, meteorology, site characteristics, and central site concentrations.

**Results:**

PM_2.5 _was strongly associated with the central site monitor (R^2 ^= 0.68). Additional variability was explained by total roadway length within 100 m of the home, smoking or grilling near the monitor, and block-group population density (R^2 ^= 0.76). EC showed greater spatial variability, especially during winter months, and was predicted by roadway length within 200 m of the home. The influence of traffic was greater under low wind speed conditions, and concentrations were lower during summer (R^2 ^= 0.52). NO_2 _showed significant spatial variability, predicted by population density and roadway length within 50 m of the home, modified by site characteristics (obstruction), and with higher concentrations during summer (R^2 ^= 0.56).

**Conclusion:**

Each pollutant examined displayed somewhat different spatial patterns within urban neighborhoods, and were differently related to local traffic and meteorology. Our results indicate a need for multi-pollutant exposure modeling to disentangle causal agents in epidemiological studies, and further investigation of site-specific and meteorological modification of the traffic-concentration relationship in urban neighborhoods.

## Background

There is a growing body of literature linking geographic information system (GIS)-based measures of traffic exposure to asthma and other respiratory outcomes. In the U.S. and Europe, children living or attending school near truck routes and highways show greater asthma symptoms [1-3], asthma hospitalizations [4,5], respiratory illness [1], allergic rhinitis [6], and reduced lung function [7]. However, proximity measures can represent a variety of pollutants or other near-roadway exposures (i.e., noise, poverty). There is no consensus on which traffic indicators may best capture variability in different pollutants within different settings, and the specific exhaust components responsible for health effects remain unidentified. For these reasons, there is a need to distinguish the relative spatial patterns of multiple traffic-related air pollutants, and to estimate concentrations using different GIS-based traffic indicators applicable across larger epidemiological studies.

Pollutants of interest include nitrogen dioxide (NO_2_), fine particulate matter (PM_2.5_), and elemental carbon (EC); each has been linked to both respiratory health and vehicular emissions. One recent study distinguished their relative spatial distributions within urban settings using GIS; this study found greater intra-urban variability and stronger traffic influences for NO_2 _and EC than for PM_2.5 _in European cities [8]. Comparable multi-pollutant analyses in the United States or in other settings have been limited, especially with a focus on residential settings within epidemiological investigations.

Addressing multiple pollutants at residences in large cohort studies is valuable but imposes constraints on the exposure assessment. For example, equipment-intensive multi-pollutant sampling (including the indoor environment) generally limits the number of sites that can be sampled simultaneously, reinforcing the need for models with spatial and temporal components, which can calibrate spatial models over time. For outcomes like asthma etiology, models estimating long-term exposures are needed, implying that measurements need to be taken at multiple points in time and that models need to separate spatial from temporal factors to the extent possible. This necessitates a careful evaluation of the role of meteorology in modifying the relationship between traffic and concentrations, a topic that has received little attention in GIS-based models to date.

In addition, issues regarding choice of traffic indicators and spatial-temporal separation may be exacerbated within urban neighborhoods, as predictors shown to be significant elsewhere (i.e., land use type) lack adequate variability to predict concentrations. Moreover, unlike measurements collected in open spaces near major roadways, residential measures may be impacted by near-home sources (e.g., idling cars, home heating, smoking, grilling) and site characteristics altering the traffic-concentration relationship (e.g., building configuration). Monitoring at residences imposes logistical constraints related to site configuration (e.g., availability of power supply, secure space), which may modify the traffic-concentration relationship. Traffic data quality can also be poor in residential areas, as most government and commercially-available datasets relay on estimates for smaller roads, reducing variability and producing significant misclassification within residential areas. Finally, in North America, the diesel fraction of total traffic in residential neighborhoods is generally smaller than in Europe, and is poorly characterized, such that total traffic measures may be less predictive of EC concentrations.

Land-use regression (LUR), a standard approach for predicting pollutant concentrations using concentration measures, GIS-derived spatial parameters, and site characteristics, allows for the characterization of exposure differentials within urban areas, and has been shown to better capture small-scale intra-urban variability than does kriging, integrated meteorological-emission (IME) models, or dispersion models [9]. LUR models of traffic-related pollution have shown stronger relationships with children's respiratory outcomes than have simple distance-to-roadway measures [10]. LUR and other GIS-based methods, however, have shown poor generalizability, as parameters selected using available spatial data and local characteristics in one city may not produce comparable estimates for another. Most LUR studies to date have been based on measurements collected near roadways or in other unobstructed urban locations, often at a fixed height [11,12]. As such, most LUR studies find a clear effect of traffic, potentially over-estimating the influence of traffic on average personal exposures in the urban area, and few LUR studies have attempted to characterize the near-home environment in dense residential areas. A recent LUR study captured a similar geographic region as our analysis, but focused on metropolitan-scale variability for a single pollutant (black carbon) with comparatively little exploration of spatial predictors, traffic terms, or meteorological contributors beyond wind speed [13]. Only one previous LUR study, to our knowledge, has attempted to account for wind speed and direction in detail, but again for a single pollutant without an explicit residential focus [14].

In this study, we used LUR techniques and GIS-derived variables to investigate the varying associations between multiple traffic indicators and outdoor residential concentrations of multiple air pollutants within the urban neighborhoods in and adjacent to Boston, Massachusetts. We evaluated a suite of GIS-based traffic indicators, and explored meteorology and residential site characteristics as potential modifiers of the traffic-concentration relationship, with the goal of understanding the extent to which traffic-concentration relationships may be different by pollutant, and ultimately to inform exposure modeling for future epidemiological analyses focused on urban cohorts.

## Methods

### Site selection

**Figure 1:**
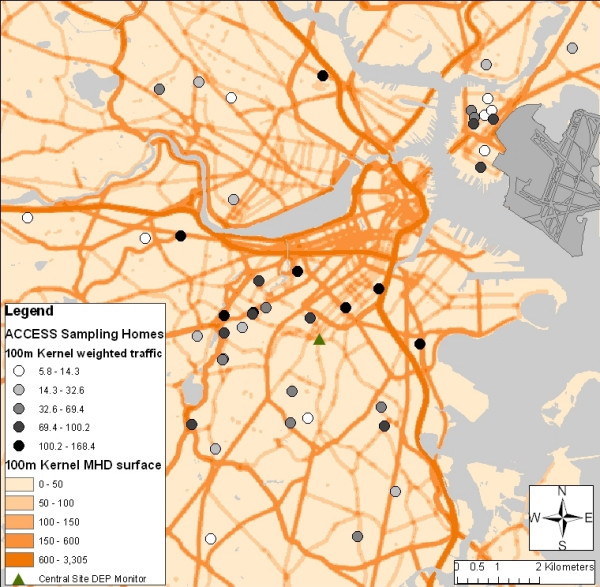
100-meter kernel-weighted traffic scores for urban area and sampling homes (Vehicle-miles per day/km^2^).

This exposure modeling effort was nested within the Asthma Coalition on Community, Environment and Social Stress (ACCESS) birth cohort study. Sample homes were selected to represent variability in traffic densities across Boston and other proximate neighborhoods. Candidate homes were geocoded using U.S. Census TIGRE files and City of Boston street parcel data, and initial traffic scores for each home were assigned using Massachusetts Highway Department (MHD) traffic volume data. As we anticipated first-order (Gaussian) decay of key pollutants in the first 100–300 meters near major roadways [15], we opted to create initial traffic scores for site selection by applying a kernel weighting function to total traffic counts for all road segments within 100 meters of the home. The kernel function approximates concentration gradients expected under Gaussian decay, assigning higher weights to road segments nearer to the home. Resultant traffic scores were divided into tertiles, and sampling homes were selected to represent the observed range of traffic scores and neighborhoods. Due to unbalanced cohort recruitment in the study's early stages, additional non-cohort participants were recruited to capture a wider range of traffic scores, and neighborhoods where further recruitment was anticipated were over-sampled. The spatial distribution of our final sampling cohort is shown in Figure 1, where homes are shaded by 100-meter kernel-weighted traffic score, against a surface of the same measure for each 50-meter cell.

### Sampling methods

We measured indoor and outdoor concentrations of PM_2.5_, NO_2_, and EC in two seasons (summer: May through early October, winter: December through March) at 44 homes across Boston and nearby urban communities, though only outdoor measures are included in this analysis. PM_2.5 _was measured using the Harvard Personal Environmental Monitor (PEM) [16], EC using reflectance analysis of PM_2.5 _filters, using the M43D Smokestain Reflectometer (Diffusion Systems Ltd., London UK) and with the absorption coefficient calculated in accordance with ISO 9835, as described in [17]. NO_2 _was analyzed using Yanagisawa passive filter badges [18], analyzed by spectrophotometry. Integrated measures for each pollutant were collected for one week per season per home wherever feasible, in two sessions of 3 to 4 days duration, averaged to one mean concentration per home per season for our LUR analysis. 24-hour traffic counts were collected using the Trax I Plus (JAMAR Technologies, Horsham, PA), on the highest-density road within 100 m of the home, during each sampling period when this was feasible (i.e., not during periods with snow/ice on the ground). Questionnaires were administered to identify nearby sources and sampling week activities that may influence concentrations, as detailed elsewhere [19].

### Additional data sources

#### Traffic Data

Road networks and traffic data were obtained from MHD. Because different aspects of traffic including density, roadway configuration, and average vehicle speed may affect emission rates, pollutant mix, and dispersion, we opted to create a suite of 25 traffic indicators (Table 1) capturing varying aspects of traffic. We built raster-based cumulative density scores for average daily traffic (ADT) counts within radii of 50 to 500 meters around each home. Because roadway segments nearer to the home may have greater influence on concentrations, we also explored inverse-distance quadratic functions (kernel-weighted buffers) for the same radii. As traffic counts on smaller residential roads were sparse, we created cumulative density scores including only larger roads (above 8,500 cars/day), summary measures of total roadway length within radii of 50 to 500 meters, and the product of roadway length and average daily traffic counts within 200 meters. We considered distance to various roadway types, including the nearest larger road (greater than 8,500 cars/day), major road (13,000 cars/day), highway, and designated truck route. Lastly, to explore the influence of major roads on nearby neighborhoods, we created indicators of its average daily traffic, diesel traffic (estimated using axle length by the Trax I Plus), and weighted each by the home's distance to the road.

**Table 1:** Traffic indicators examined for GIS-based LUR models.

**Indicator type**	**Indicator**	**Units**
**Cumulative density scores:**	Unweighted density within: 50, 100, 200, 300, 500 m buffers	Vehicle-meters/per day/m^2^
	Kernel-weighted density: 50, 100, 200, 300, 500 m buffers	Vehicle-meters/per day/m^2^
	Density of urban roads (> 8500 cars/day) within 200 m	Vehicle-meters/per day/m^2^
**Summary measures:**	Total roadway length within: 50, 100, 200, 300, 500 m	Meters
	Total ADT*Length (VMT) within 200 m	Vehicle-meters per day
**Distance-based measures:**	Distance to nearest urban road (>8500 cars/day)	Meters
	To nearest major road (>13,000 cars/day)	Meters
	To nearest highway (>19,000 cars/day)	Meters
	To nearest MHD-designated truck route	Meters
**Characteristics of nearest major road:**	Average daily traffic (ADT)	Vehicles/day
	ADT/Distance to major road	(Vehicles/day)/m
	Diesel fraction	Percent (%)
	Trucks per day	Vehicles/day
	Trucks/Distance to major road	(Vehicles/day)/m

We considered other GIS covariates that may be associated with traffic, represent other pollutant sources, or modify the observed traffic-concentration relationship. Block group-level population and area measures were used to estimate population density. NCLD-50 land use categories and elevation data were downloaded from the USGS National Land Cover Dataset (NLCD) and National Elevation Dataset (NED), respectively.

#### Temporal variability: Background concentrations and meteorology

With a residential multi-pollutant approach, we were able to sample at a maximum of three homes per week, creating the need to account for temporal variability in background concentrations and meteorology. We estimated the influence of temporal heterogeneity in our data by regressing measured concentrations against mean central site concentrations for specific hours that each sample was collected. This temporal correction method is similar to that used elsewhere [20], though annual averages were not calculated.

Our primary central site concentration data were collected from the Massachusetts Department of Environmental Protection (DEP) monitor in the central Roxbury neighborhood (Figure 1). Hourly NO_2 _is measured at the DEP monitor using the TECO42c chemiluminescence method. Hourly PM_2.5 _is measured using the Met-One BAM with a PM_2.5 _SCC beta attenuation method. Notably, EC is measured using the AE22ER aethelometer for optical absorption (Magee Scientific, Berkeley CA), as compared with the reflectance analysis used at our homes. The relationship between aethelometer and reflectance measures of EC has previously been found to differ by season in Boston, with aethelometers reading high in the summer (biased upward by 30% or more) and lower in winter (G. Allen, personal communication), and recent studies have shown similar seasonal biases with the aethalometer in other cold weather settings [21]. Although hourly data NO_2 _were available at two additional nearby sites, with hourly data for EC and PM_2.5 _available at one additional site each within the city, we used the Roxbury central site monitor in our main model given the availability of all three pollutants. We regressed outdoor PM_2.5_, EC, and NO_2 _concentrations against mean DEP concentrations for the specific hours that each residential sample was collected. Temporally-adjusted residuals were used for selection of spatial covariates, and final models were sensitivity tested against the use of data from other DEP monitors.

Meteorological data were collected from the same central site, because windspeed and direction could not be measured at each home during the sampling period. Mean windspeed and direction were calculated for daytime hours (6am–9pm) within each sampling period, when we anticipate significant traffic, our main source of interest. Further, several wind parameters were created in relation to traffic sources (i.e., percent of sampling hours when home is downwind from the nearest road), such that significance of the wind term implies source significance. Lastly, although meteorological texts define 'still winds' as below 1 m/s [22], we used 2.0 m/s to better dichotomize our high-windspeed dataset (median = 4.9 m/s). Meteorological factors and other covariates considered as effect modifiers of the traffic-pollution relationship are summarized in Table 2.

**Table 2:** Potential effect modifiers of traffic-concentration relationship

		**Units**
**Home characteristics:**	Obstructed from road	Yes/no
	Obstructed from major road	Yes/no
**Sampling period characteristics:**	Percent of hours downwind from major road	Percent (%)
	Average windspeed during daytime sampling hours (6am–9pm)	m/s
	Percent daytime hours with still winds (< 2 m/s)	Percent (%)
	Percent of weekend sampling days	Percent (%)
	Floor (monitor height)	(Categorical: 1, 2, 3+)
	Snow during sampling period	Yes/no

#### Analytic methods and model-building

We built models separately by pollutant, allowing different aspects of traffic, meteorology, and site-specific factors to predict concentrations of different pollutants. We selected candidate traffic indicators and modifiers against the temporally-corrected residuals, using nonparametric univariate correlations (Spearman correlations, p < 0.3) of concentrations against traffic indicators as our primary selection method.

Because traffic indicators are highly correlated, however, we considered cluster analysis as a secondary selection method; the tree command in R groups observed concentrations by applying an impurity criterion to minimize within-group variances while maximizing between-group differences. The command compared concentration groups created using the 25 examined traffic indicators as predictors, and returned the indicators which best distinguished, as a group, high and low pollution locations, and the most effective binary cut-point for each indicator. Multivariate models were built using those traffic indicators selected by both correlation and clustering methods.

Using a stepwise forward regression process, we first included central site data, then traffic indicators, meteorological and site-specific modifiers as interaction terms with traffic indicators. Finally, we examined the effect of additional sources (e.g., grilling or smoking noted near outdoor monitor, block group population density, land use type, proximity to industry, season). We note that several of these indicators may be associated with traffic, capturing some traffic effect. We used the general form of Equation 1, and a maximum p-value of 0.1 to retain variables at each stage.

(1) Concentration_ijt _= β_0j _+ β_1j _*DEP_jt _+ β_2j _*Traffic_i _+ β_3j _*Traffic_i _*Modifier_it _+ β_4j _*Other sources_it _+ e_ijt_

Where Concentration_ijt _is the measured concentration of pollutant *j *at location (home) *i *during sampling period (time) *t*. DEP_jt _is the mean concentration of pollutant *j *at the central site during sampling period *t*. Traffic_i _is the value of each traffic indicator listed in Table 1, tested separately in prediction models, at location *i*. Modifier_it _is the value of meteorological or site characteristics altering the association between traffic indicators and Concentration_ijt_. PM_2.5_, EC, and DEP_jt _values for PM_2.5 _and EC were log-distributed, and thus transformed prior to covariate selection and model building in our primary model. NO_2 _values were normally distributed, and not transformed.

For residential EC, reflectance values are indicated by filter absorbance (units of m^-1 ^*10^-5^). To facilitate interpretation of our findings, we approximate these values to as mass units using 0.83 μg/m^3^/m^-1 ^*10^-5^, derived from side-by-side reflectance and quartz fiber samples collected during summer in the urban northeastern U.S. [23]. This relationship may vary by location and season; because we anticipated that residential EC may display a different relationship with central site EC by season, we built our models using non-converted (reflectance) units, and allowed for season-specific slopes in each model. Along with the aforementioned concerns about biases with aethalometer data and seasonal variability in the mass-absorption relationship, hypothesized sources of EC (i.e., wood smoke, home heating fuel) may display greater spatial variability during winter, when lower atmospheric mixing height may increase their influence.

#### Sensitivity Analyses

Extensive sensitivity tests were performed on the final model for each pollutant. Models were examined for sensitivity to the selection of traffic indicator by individually substituting each traffic indicator from Table 1. Likewise, we examined the selection of meteorological and site-specific modifiers by individually substituting other candidates. In each case, the final model was retained based upon overall model fit (R^2^).

To examine the quality of resolution in our area-level (raster) GIS data, we considered a range of base cell sizes (the smallest spatial unit employed in variable creation), varying in width from 10 to 50 meters. To test the quality and robustness of our road traffic data, we compared our traffic indicators derived from MHD data to comparable indicators using three other data sources. We initially log-transformed PM_2.5 _and EC data due to its lognormal concentration distribution, and tested the effect of this transformation. To examine the potential for residual spatial confounding using our central-site monitor data, we evaluated the use of other Boston-area DEP monitors. To assess residual seasonality not captured by DEP data, we tested a categorical season term and seasonally-varying slopes associating home data to the central site for all pollutants. Finally, we examined the robustness of each model to within-site autocorrelation owing to multiple measures at each site, using random effects by household.

All traffic and land use variables were created in ArcGIS 9, clustering analyses were performed using the tree command in R version 2.2.0, and model-building in SAS version 9.1.

## Results

We conducted 66 sampling sessions in total, consisting of 86 three-to-four day measurements in 44 homes. Fifty-one measurements were taken in 36 homes during summer months, and 35 measurements were taken in 25 homes during winter months. Table 3 summarizes the within-season average concentrations by sampling session for each pollutant. PM_2.5 _and EC were significantly correlated during winter and summer (p < 0.05), while EC and NO_2 _were marginally correlated in both seasons, and PM_2.5 _and NO_2 _were not.

**Table 3:** Within-season average outdoor concentrations at homes and central-site monitor

		**Overall**			**Summer**			**Winter**		
**Pollutant**	**Location**	**N**	**Mean (SD)**	**Median**	**N**	**Mean (SD)**	**Median**	**N**	**Mean (SD)**	**Median**
**PM**_2.5_	Outdoor	59	13.9 (5.0)	12.5	35	15.1 (5.7)	13.8	24	12.2 (3.1)	13.8
(μg/m^3^)	Central Site	59	15.4 (6.1)	14.6	35	17.0 (7.1)	14.9	24	13.1 (3.4)	12.9
**EC**	Outdoor	58	0.52 (0.41)	0.46	34	0.51 (0.51)	0.39	24	0.54 (0.18)	0.56
(μg/m^3^)	Central Site	58	0.86 (0.34)	0.83	34	1.01 (0.31)	1.01	24	0.65 (0.27)	0.61
**NO**_2_	Outdoor	52	17.2 (6.0)	16.8	36	17.7 (6.4)	17.3	16	16.3 (4.9)	15.5
(ppb)	Central Site	52	18.4 (3.9)	17.9	36	17.3 (3.5)	18.0	16	21.0 (3.4)	21.3

### Pollutant-specific modeling results

**Figure 2:**
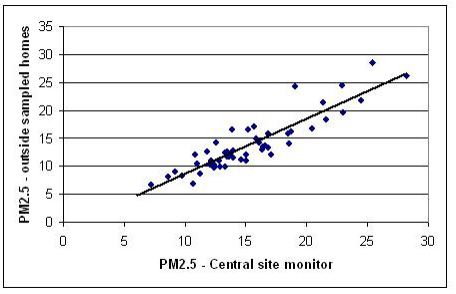
**Scatter plots of outdoor concentrations vs. central site concentration averages during sampling periods.** PM_2.5 _at homes vs. central site (μg/m^3^).

Outdoor PM_2.5 _was highly correlated with central-site PM_2.5 _(R^2 ^= 0.68), as suggested in Figure 2, indicating a predominance of temporal variability and relative spatial homogeneity in PM_2.5 _across the urban area. In multivariate regressions including central site data, the best traffic indicator was total roadway length within 100 meters of the home (Table 4). Final multivariate model results indicate that the traffic-PM_2.5 _relationship was not significantly altered by any of our candidate modifiers. Other combustion sources (smoking or grilling) and population density significantly contributed to concentrations (overall R^2 ^= 0.76).

**Table 4:** Final model results for three pollutants

		**ln(PM**_2.5_**) **(μg/m^3^)			**ln(EC) **(m^-1^*10^-5^)			**NO**_2 _(ppb)	
**Predictor Type**	**Model**	β (p-value)	**Sequential R**^2^	**Model**	β (p-value)	**Sequential R**^2^	**Model**	β (p-value)	**Sequential R**^2^
**Intercept**		0.205 *(.32)*	--		-0.907 *(<.0001)*	--		-12.50 (*.009)*	--
**Central site Concentration**	ln (Central Site [PM_2.5_])	0.776 *(<.0001)*	.68	ln (Central site [EC])	0.103 *(.59)*	.03	Central site [NO_2_]	1.06 *(<.0001)*	.21
				ln (Central site [EC]) * warmer season	0.82 (.004)	.26			
**Traffic Indicator**	Roadway Length in 100 m	1.48*10^-4 ^*(.02)*	.70	Roadway Length in 200 m	1.10 * 10^-4 ^*(.01)*	.40	Roadway Length in 50 m	0.0144 *(.002)*	.22
Traffic Indicator* Modifier	N/A	N/A	N/A	Roadway Length in 200 m × % Hours of Still Winds	4.38 *10^-4 ^*(.02)*	.48	Roadway Length in 50 m × Obstructed Major Rd	-0.0094 *(.005)*	.31
**Other Sources/Land Use**	Smoking or grilling	0.156 *(.01)*	.73	Warmer Season	-0.268 *(.057)*	.52	Warmer Season	4.93 *(.001)*	.44
	Population Density	9.24*10^-6 ^*(.01)*	.76				Population Density	4.01*10^-4 ^*(.001)*	.56

**Figure 3:**
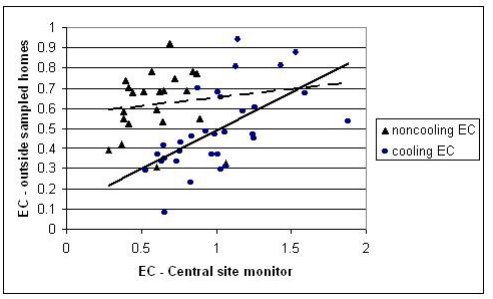
**Scatter plots of outdoor concentrations vs. central site concentration averages during sampling periods.** EC at homes vs. central site (μg/m^3^); one influential point removed each season.

EC shows relatively poor associations with central site data overall (R^2 ^= 0.08), though this is partly attributable to seasonal differences in the relationship (Figure 3), with varying slopes and stronger correlations during summer (Spearman r = 0.66) than winter (r = 0.37). In the final multivariate model (R^2 ^= 0.52), EC was best predicted by total roadway length within 200 meters of the home, and the association between EC and traffic was increased under low wind speed conditions. During summer months, residential EC concentrations were somewhat lower and displayed stronger associations with central site data. Approximately 30% of the variability in EC was explained by temporal terms, and 14% by the traffic term (spatial component). The interaction of traffic with hours of low wind speed, incorporating both spatial and temporal variance, accounted for an additional 8%.

**Figure 4:**
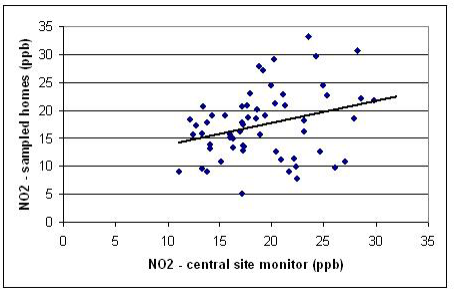
**Scatter plots of outdoor concentrations vs. central site concentration averages during sampling periods.** NO_2 _at homes vs. central site (ppb).

NO_2 _was weakly associated with central site concentrations (R^2 ^= 0.21), suggesting significant spatial heterogeneity within urban residential areas (Figure 4). The final multivariate model (R^2 ^= 0.56) includes total roadway length within 50 meters of the home, significantly attenuated by an obstruction (i.e., building) between the monitor and nearest major road. Residential NO_2 _concentrations were higher during summer months, and positively associated with population density (Table 4). Spatial terms (traffic, obstruction between the monitor and nearest major road, and population density) together account for approximately 23% of NO_2_variability. Temporal terms (central site, summer months) account for about 34%.

### Sensitivity analyses

#### Selection of traffic indicator

Sensitivity analyses indicate that other traffic indicators could not be substituted to create a comparable model for PM_2.5 _(Table 5) For EC (Table 6), diesel-based measures can explain more variability, with R^2 ^values of approximately 0.54, but were available for only a subset of locations (n = 34) and thus were not considered robust for the primary model. For the full cohort, no indicator was exchangeable with roadway length within 200 meters of the home. In addition, the interaction term of traffic modified by low wind speeds remained significant in several cases where the main effect of traffic did not maintain significance. For NO_2_, sensitivity tests (Table 7) support the finding that shorter buffer lengths were most effective. Larger buffer lengths did not produce a comparable model, but kernel-weighted traffic density within 50 meters of the home could be substituted effectively, as could unweighted cumulative density within 100 meters.

**Table 5:** Sensitivity analyses including best predictor in each category, all summary measures.

**Indicator Type**	**Traffic Indicator**	**Estimate(s), p-value(s) from multivariate model**	**Model R**^2^
**Base model (w/out traffic)**			R^2 ^= .74
**Cumulative Density scores**	Unweighted 500 m traffic density (n = 57)	β1 = 4.74*10^-4 ^(.17)	R^2 ^= .75
**Summary Measures**	Total roadway length:		
	50 meters (n = 57)	β1 = 1.31*10^-4 ^(.35)	R^2 ^= .74
	**100 meters (n = 57)**	β1 = 1.48*10^-4^**(.02)**	**R**^2^**= .76**
	200 meters (n = 57)	β1 = 1.42*10^-5 ^(.56)	R^2 ^= .74
	300 meters (n = 57)	β1 = 5.54*10^-6 ^(.57)	R^2 ^= .74
**Distance-based measures**	Distance to nearest designated truck route (n = 57)	β1 = 1.80*10^-5 ^(.62)	R^2 ^= .74
**Characteristics of nearest major road**	Average daily traffic (ADT) (n = 57)	β1 = -3.04*10^-6 ^(.25)	R^2 ^= .74

Final model bolded; models with diesel-based terms italicized. **Traffic indicators for PM**_**2.5**_

**Table 6:** Sensitivity analyses including best predictor in each category, all summary measures.

**Indicator Type**	**Traffic Indicator**	**Estimate(s), p-value(s) from multivariate model**	**Model R**^2^
**Base model**			R^2 ^= .31
**Cumulative density scores:**	Unweighted 500 m buffer (n = 54)	β1 = 5.39*10^-4 ^(.49) β2 = 5.63*10^-3 ^(.41)	R^2 ^= .39
**Summary measures:**	Total roadway length within:		
	50 meters *Still Winds (n = 54)	β1 = 3.94*10^-4 ^(.18) β2 = 4.08*10^-3 ^(.01)	R^2 ^= .41
	100 meters *Still Winds (n = 54)	β1 = 2.15*10^-4 ^(.14) β2 = 1.27*10^-3 ^(.03)	R^2 ^= .47
	200 meters *Still Winds (n = 54)	β1 = 1.10*10^-4^(.01) β2 = 4.38*10^-4^(.02)	**R**^2^**= .52**
	300 meters *Still Winds (n = 54)	β1 = 2.99*10^-5 ^(.11 β2 = 2.01*10^-4 ^(.04)	R^2 ^= .48
Distance-based measures:	To nearest highway (>19,000 cars/day) * Still Winds (n = 54)	β1 = 0.452 (.06) β2 = 0.549 (.04)	R^2 ^= .45
**Characteristics of Nearest major road:**	Diesel fraction * Still Winds (n = 34)	*β1 = -1.06 (.02)* *β2 = 34.6 (.02)*	*R*^2^*= .54*
	Trucks per day * Still Winds (n = 34)	β1 = -7.41*10^-5^*(.06)* β2 = 3.51*10^-3^*(.02)*	*R*^2^*= .54*
	Trucks/Distance to major road * Still Winds (n = 34)	β1 = -6.31*10^-3^*(.03)* *β2 = s0.119 (.05)*	*R*^2^*= .54*

Final model bolded; models with diesel-based terms italicized. **Traffic indicators for EC**.

**Table 7:** Sensitivity analyses including best predictor in each category, all summary measures.

**Indicator Type**	**Traffic Indicator**	**Estimate(s), p-value(s) from multivariate model**	**Model R**^2^
**Null model**			R^2 ^= .39
**Cumulative density scores:**	Unweighted traffic density in 100 m * Obs nearest Major Road (n = 50)	β1 = 0.055 (.004) β2 = -0.051 (.004)	R^2 ^= .55
	Kernel-weighted density in 50 m * Obstructed (n = 50)	β1 = 0.034 (.02) β2 = -0.056 (.002)	R^2 ^= .55
	Density of urban roads (>8500 cars/day) in 200 m * Obstructed (n = 50)	β1 = 589.4 (.049) β2 = -760.9 (.0095)	R^2 ^= .52
**Summary measures:**	Total roadway length within:		
	50 meters * Obstructed (n = 50)	**β1 = 0.0144 (<.0001)** **β2 = -0.0094 (.005)**	**R**^2 ^**= .56**
	100 meters * Obstructed (n = 50)	β1 = 0.0022 (.34) β2 = -0.0042 (.005)	R^2 ^= .54
	200 meters * Obstructed (n = 50)	β1 = 9.28*10^-4 ^(.22) β2 = -1.25*10^-3 ^(.008)	R^2 ^= .52
	300 meters * Obstructed (n = 50)	β1 = 2.31*10^-4 ^(.55) β2 = -6.65*10^-4 ^(.0095)	R^2 ^= .50
	Vehicle Miles Traveled in 200 m * Obstructed (n = 50)	β1 = 1*10^-7 ^(.21) β2 = -1*10^-7 ^(.10)	R^2 ^= .47
**Distance-based measures:**	To nearest highway (>19,000/day) * Obstructed (n = 50)	β1= 0.0176 (.12) β2 = -0.0195 (.07)	R^2 ^= .50
**Characteristics of nearest major road:**	Trucks per day on nearest major road * Obstructed (n = 34)	*β1 = .0061 (.01)* *β2 = .00596 (.01)*	*R^2 ^*= *.59*

Final model bolded; models with diesel-based terms italicized. **Traffic indicators for NO**_**2**_.

#### Accuracy of traffic data

To validate raster-based traffic indicators, we considered a range of base cell sizes from 10 to 50 meters square, bearing little difference on traffic indicator values compared to our default 25 meter cell size. Given concerns about data quality, where possible we verified MHD counts against traffic data obtained from the Massachusetts Executive Office of Transportation, ESRI Business Analyst, and our traffic counts collected outside cohort homes using the Jamar Trax I device. Correlations across traffic sources were generally above 0.7.

#### Selection of central site monitor

We considered several alternatives to the use of the Roxbury central site monitor concentrations for temporal correction, including using the other available urban monitors individually, the average concentration from all urban monitors available during each sampling period, and the mean concentration at a background monitor south of Boston (available for summer months only). No alternative to the Roxbury central site sampling period mean explained greater variability in concentrations or significantly altered traffic-pollution relationships in multivariate models.

#### Selection of meteorological and site-specific modifiers for EC and NO_2_s

All EC models showed a significant, positive effect of low wind speeds on the traffic-pollution relationship. Sensitivity analyses indicated that other wind variables (mean daytime windspeed, percent of day downwind from road) were significant and may be substituted for percent of low wind speed hours, losing only marginal explanatory power (R^2 ^= 0.52 and 0.49, respectively). The similar findings for windspeed and direction may be expected, as windspeed and direction at our central site were highly correlated, with higher windspeeds from the west (data not shown).

For NO_2_, no other modifier could replace obstruction between the monitor and nearest major road in the final model. Because presence of an obstruction could theoretically proxy for distance to nearest major road, we replaced the term with distance to major road, and found highly non-significant results, indicating that this was not likely the case.

#### Log-transformation of PM_2.5 _and EC data

The selection of the 100-meter roadway length term and other predictors for the PM_2.5 _model was not dependent on log transformation. Using un-transformed PM_2.5_, we achieve an R^2 ^of 0.73, and retain significance in all predictors. For un-transformed EC, the same traffic term and all other predictors retained significance, with an R^2 ^of 0.51.

#### Inclusion of a categorical variable for season

Because the season term may be extraneous in models including temporal data from a central site, we explored the effect of removing this term from the final EC and NO_2 _models. For EC, removing the season term caused the central site monitor estimate to drop by half and fall out of significance, while the effect of low wind speed increased by almost 50%, and overall model fit declined. Because of this decline in overall explanatory power when removed and the interpretability of the term given methodological aspects of EC concentration estimation, we opted to maintain the season term and season-specific slopes on the central site monitor term in the final model.

For NO_2_, dropping the season term decreased the effect of the central site monitor by approximately 50%, but did not affect overall model fit or other parameters. Thus, although NO_2 _is higher at our residences during summer months, the effect is captured in part by the central site monitor; because the term did not significantly alter other parameters, we opted to leave it in the final model. Finally, we tested the addition of a season term to the PM_2.5 _model, and found no effect on the central site term or overall fit, although the influence of other combustion sources (i.e., smoking or grilling) was increased by approximately 35%, indicating possible seasonal differences in source activities. Because traffic is our main source of interest, however, and because this result did not improve overall fit or alter the observed influence of traffic, we opted to maintain the more parsimonious original PM_2.5 _model.

#### Robustness to within-site autocorrelation

For all pollutants, because the majority of homes were monitored in two seasons, we examined the effect of within-site autocorrelation using random effects by household. Autocorrelation by site did not influence model fit or parameter estimates for any model.

Finally, a one-at-a-time exclusion cross-validation was performed to assess the internal consistency of model results. The Spearman correlation between estimated and measured log PM_2.5 _was 0.80, 0.66 for log EC, and 0.66 for NO_2 _(p < .0001 in all cases), indicating strong associations between predicted and actual values, indicating acceptable internal validity.

## Discussion

Working strictly within urban neighborhoods and employing a multi-pollutant approach, our study offers several findings useful to future research exploring and modeling air pollution exposures for epidemiological purposes. These observations broadly apply to four areas: (1) urban residential variability in traffic densities and pollution concentrations, (2) fraction of urban residential pollution that is attributable to traffic, (3) selection of traffic indicators for residential exposure estimation, and (4) modification of traffic-concentration relationships by site characteristics and meteorology.

### (1) Urban residential variability in traffic densities and pollution concentrations

We found significantly greater variability and stronger relationships with local traffic for EC and NO_2 _than for PM_2.5_, consistent with prior literature [24,25], and which corroborates evidence that PM_2.5 _patterns are largely regional in nature for the Eastern U.S. [26,27]. We found somewhat weaker correlations across the three pollutants than have been shown in prior European studies [1], potentially because of our focus within urban neighborhoods relatively proximate to one another, while most prior intra-urban studies have actually examined metropolitan regions.

Though we found significant variability in traffic density across cohort homes, traffic varied somewhat less within residential neighborhoods than across the entire urban core, as shown in Figure 1. Across the entire urban core, 100-meter kernel-weighted traffic scores ranged from 0 to 3,305 vehicle miles traveled (VMT) per m^2 ^per day; at cohort homes, this measure ranged from 5.8 to 168 VMT/m^2^-day. This difference is driven largely by major highways, alongside which relatively few homes are located, but this observation may be important for exposure estimation; many models are derived from concentration data near major roads, which may inaccurately reflect traffic-concentration associations at the lower end.

### (2) Fraction of urban residential pollution that is attributable to traffic

Using the traffic-pollution relationship observed across our sampling sites, we can estimate the portion of residential concentrations that are attributable to traffic. For PM_2.5_, the mean 100-meter summary roadway length of 1,110 meters accounted for a marginal contribution of 1.2 μg/m^3^, or 9.7% of predicted PM_2.5_. Applying our predictive models with mean values for all terms, mean predicted concentration is 13.2 μg/m^3^; an increase of one standard deviation in roadway length alone (371 meters) increases concentrations to 13.9 μg/m^3^. Population density, which likely captures some traffic-related influence, adds 1.1 μg/m^3 ^on average. Using non-transformed PM_2.5_, the predicted combined traffic contribution is somewhat larger (2.6 μg/m^3^).

For EC, the mean 200-meter buffer roadway length of 3,560 meters accounted for approximately 0.17 μg/m^3^, or 36% of total predicted EC. Increasing roadway length alone by one standard deviation (1,156 meters), with all other parameters at their mean values, increases predictions from 0.47 to 0.54 μg/m^3^. Using non-transformed EC concentrations, total predicted traffic contributions are somewhat larger (0.39 μg/m^3^). We observed a gradient of almost 1 μg/m^3 ^in EC across sampled homes before correcting for temporal variability, which is relatively small compared to that observed in European studies (approximately 10 μg/m^3^), as expected given the prevalence of diesel passenger vehicles in Europe [28].

Modeled traffic terms accounted for approximately 2.8 ppb, or 21% of modeled NO_2_. A mean 50-meter buffer roadway length of 441 meters, with mean values for other terms, predicts a concentration of 17.8 ppb. A one standard deviation increase (179 meters) increases concentrations to 18.9 ppb. The range of 50-meter roadway lengths observed predicts a NO_2 _range of 15.4 to 20.9 ppb. Population density, likely capturing some traffic effect, and accounts for 4.4 ppb on average. The full range of NO_2 _variability observed (including spatial and temporal components) was approximately 28 ppb (53 μg/m^3^), on the order of exposures associated elsewhere with increased wheeze and cough among children [29].

### (3) Selection of traffic indicators

Total roadway length within varying buffer radii provided useful traffic indicators for each pollutant. Because actual traffic counts for residential roads are generally sparse, length measures provided more stable traffic indicators in these areas than do ADT-based estimates. There is also differential bias in traffic count accuracy by roadway size in the traffic data available for many cities; actual traffic counts are generally collected on a regular basis for highways and major roads, and rough estimates are created for smaller residential roads.

While correlated, total roadway lengths within various buffers were not interchangeable in predictive models. The 100-meter buffer length effective for PM_2.5 _coincides with our original buffer radius created for site selection, selected because maximum declines in particle concentrations occur in the first 100–300 meters alongside major roadways, dependent on particle size distributions and wind characteristics. We found a larger buffer of 200 m to be most predictive for EC; a meta-analysis of the literature [30] found buffers ranging from 50–250 meters across studies. The slightly higher value for EC than for PM_2.5 _could be related to differential contributions of background and related complications, the relative lack of diesel sources and the likelihood of such sources on major roads (indicated by the significance of diesel terms related to nearest major road), or to the smaller particle size distribution anticipated for EC. For NO_2_, shorter buffer lengths of 50 meters were most effective. Secondary formation of NO_2 _from NO might suggest longer buffer lengths, with one LUR study showing 300 m buffers to be effective [31] and one meta-analysis [30] indicating values of 200–500 m across studies. Other data, however, suggests that NOx concentrations can decline up to 60 percent, and NO_2 _by 30 percent, within the first 50 meters from a roadway [12]. More generally, the precise distance-concentration decay relationship for NO_2 _varies by setting, as dispersion, dilution, and chemical transformation are affected by the local pollution mix, wind characteristics, and meteorology.

Sensitivity tests suggest that diesel-based indicators may better predict EC and possibly NO_2_. Diesel influence raises a critical difference between traffic modeling in North America and Europe, as diesel vehicles constitute a smaller and poorly measured fraction of traffic in North American residential areas. Here, we rely on axle-length estimates from our traffic counter on one major road near the home, to approximate vehicle type (i.e., CNG-powered buses, for example, would be categorized here as "diesel"). In Europe, the higher prevalence of diesel passenger vehicles implies that total traffic may itself act as a diesel marker, providing more stable predictors for EC.

### (4) Modification of traffic-concentration relationships by site characteristics and meteorology

We found that accurate exposure modeling near urban residences required some consideration of site characteristics, such as population density and obstructions, which explained significant variability in concentrations and altered traffic-concentration associations. Population density, significant for PM_2.5 _and NO_2_, may proxy either for other residential sources or for traffic, and may indicate higher per-mile emission rates from 'stop-and-go' traffic in denser residential neighborhoods. We expected obstruction to modify PM or EC, in keeping with recent findings suggesting that roadside barriers reduce PM concentrations [32]. Though we did not expect to find this effect for a gaseous pollutant such as NO_2_, this result is supported by recent evidence that residential NO_2 _concentrations differed significantly depending on whether the home faced onto the courtyard or street, after accounting for distance to road [33]. Our findings may also be in part related to our passive sampling approach (as an obstruction could reduce the face velocity on the sampler).

## Limitations

Although our models are physically interpretable and explain significant variability, there are some limitations to our analysis. Our small sample size may be a limitation in model-building, though sensitivity analyses show our findings to be robust. Similarly, our multi-pollutant approach limited our sampling design to sample at only a few homes simultaneously and distributing sampling sessions over the course of a season, such that samples incorporate both temporal and spatial variability. Measuring at a large number of homes simultaneously, however, is generally infeasible for equipment-intensive multi-pollutant sampling designs, especially given interest in both the indoor and outdoor environment. In addition, long-term residential exposure estimation can benefit from within-season temporal variability, which can not be obtained from short-term simultaneous sampling campaigns. Further, the influence of meteorological covariates may be under-estimated using such approaches, and are needed for long-term exposure estimation models. The central site monitor used to account for temporal heterogeneity may capture some local-source component as well, and for this reason we opted to use only one central site monitor with the most complete data coverage for all three pollutants, to avoid confounding spatial and temporal variability during periods when some sites were unavailable. In addition, we chose to maintain the central site term in our final models, rather than predicting the temporally-corrected concentration residual, and included other temporal terms such as season and meteorological parameters.

Ultimately, most GIS-based residential exposure models are intended to allow exposure estimation across large cohorts, and thus we rely on readily-created GIS-based traffic indicators generally available across urban neighborhoods, such as total roadway length measures. Several predictors in our models, however, such as obstruction between the home and nearest major road, are effectively correction factors for the restrictions associated with residential monitoring – i.e. samplers often need be set up behind the buildings, wherever power sources are available, on a porch where smoking or grilling also occurred, or some such non-ideal location. These parameters may not be appropriate for extrapolation, as they may not reflect mean concentrations near the home, but are important to correctly interpreting residential data.

There are a number of issues related to LUR studies which limit model generalizability. First, LUR model results are highly dependent upon the quality of spatial data available. Here, for example, total roadway length produced the strongest concentration estimates in our urban neighborhoods. In areas with better traffic data, however, indicators incorporating traffic density may fare better. Second, spatial variables can have different meaning in different settings; in rural areas, for example, proximity to major roads may be correlated with proximity to parking lots, industrial areas, and other sources, which is less likely in urban areas. Similarly, overall traffic counts in Europe can provide better estimates for EC modeling due to the higher prevalence of diesel vehicles. Higher emissions of NO_2 _and total particle mass from diesel engines suggests that we may expect higher R^2^'s on European LUR models from intra-urban variability in traffic-related pollutants than in the US.

These issues of generalizability continue to challenge the search for "causal agents" in the association between traffic density and respiratory and cardiovascular illness. Because different traffic indicators have been shown to predict concentrations (and illness) in different regions, it is difficult to identify the specific spatial characteristics of unidentified causal agents. We maintain, however, that because the chemical and physical properties among various pollutants lead to differing rates of decay and deposition near roadways [15,30], predictive models should be built and compared for multiple pollutants in epidemiological studies of the effect of traffic exposure on health. Further, because pollutants more refined than PM_2.5 _are still complex (e.g., EC, a subset of PM_2.5_, may have VOCs and metals bound to its surface), there remains a need for spatial models investigating distributions of specific PM_2.5 _constituents to evaluate their relationship with health outcomes. Finally, most urban residents of North American cities spend the majority of their time indoors, further complicating efforts to define causal pollutants in the traffic-health relationship. The models presented here do not address infiltration or indoor residential environments directly, but do facilitate estimation of indoor exposures when combined with home characteristics such as building type [19]. Finally, measurement error is differential across the three pollutants modeled here, as evidenced by their varying R^2^'s, which complicates comparisons across predictive models for different pollutants in epidemiological studies. This issue deserves greater attention, as such comparisons across pollutant-specific models will be important in identifying causal agents.

Our results provide some insight to researchers working to elucidate intra-urban residential exposures for long-term epidemiological analyses. First, the issue of distinguishing temporal from spatial variability is a significant difficulty for multi-pollutant sampling designs; when only a small number of homes can be sampled simultaneously, there is not adequate variability observed within any time period to distinguish the temporal from spatial effects. To this end, we suggest, in cities lacking a year-round rural background monitor for all pollutants of interest, a fixed-site monitor (using identical methods to those at the homes) in a less heavily-trafficked area would be useful to capture background concentrations and long-range transport. We also suggest exploration of sampling designs which maximize temporal overlap; for example, a systematically-staggered sampling with a predictable amount of overlap at different homes might allow for a simplified temporal correction term. This design would retain intra-season variability as well, which is lost in the shorter-term simultaneous sampling campaigns. However, such theoretically optimal sampling designs should be approached realistically, with the recognition that residential sampling will require some amount of visit rescheduling (especially for a cohort such as ours, with women who are pregnant or with young children). Finally, in selecting sampling locations, certain site characteristics potentially impeding accurate sample collection should be considered (e.g., construction, lack of secure outdoor space or power outlets), and incorporated into previous methods for optimizing concentration variability, such as that outlined in [12].

## Conclusion

Our analysis explored a range of GIS-based traffic indicators to capture small-scale variability in multiple air pollutants within dense urban neighborhoods. Because our measures were collected outside residential homes rather than at roadside locations, our measures likely reflect more realistic exposures to urban residents. The resultant sampling design, however, raised a number of methodological challenges, including the need to account for spatial and temporal variability in measures collected during different weeks, and the need to account for home site characteristics and other residential sources which may obscure the true traffic-concentration relationship.

We found that traffic indicators not reliant on ADT estimates (i.e. roadway lengths) provided more stable predictors in the residential settings in our study. As shown elsewhere, greater spatial variability was observed in NO_2 _and EC than in PM_2.5_, and LUR techniques worked well within the urban setting to capture pollutant variability, although parameters useful at the metropolitan scale (e.g., land use type) displayed low variability and limited predictive power. Finally, the relationship between traffic and pollution concentrations was significantly modified by meteorological factors and site characteristics, indicating the importance of incorporating small-scale spatial and temporal predictors to accurately capture exposure variability in urban residential settings.

## List of abbreviations

ACCESS: Asthma Coalition on Community, Environment, and Social Stress; ADT: average daily traffic; CNG: compressed natural gas; DEP: Massachusetts Department of Environmental Protection; EC: elemental carbon; GIS: geographic information systems; IME: integrated meteorological-emission models; LUR: land use regression; MHD: Massachusetts Highway Department; NED: National Elevation Dataset; NLCD: National Land Cover Dataset; NO_2_: nitrogen dioxide; PEM: Harvard Personal Environmental Monitor; PM_2.5_: fine particulate matter, smaller than 2.5 microns mean diameter; USGS: United States Geological Survey; VMT: vehicle miles traveled.

## Competing interests

The authors declare that they have no competing interests.

## Authors' contributions

JEC participated in exposure study design, sampling design, and data collection, created all spatial and meteorological variables, performed statistical analyses, and drafted the manuscript. RJW oversaw cohort recruitment and overall ACCESS study design. LKB participated in sampling design, data collection, and analysis. JIL oversaw sampling design, data collection, data analysis, and manuscript development. All authors read and approved the final manuscript.
